# Dietary choline intake and health outcomes in U.S. adults: exploring the impact on cardiovascular disease, cancer prevalence, and all-cause mortality

**DOI:** 10.1186/s41043-024-00528-0

**Published:** 2024-05-06

**Authors:** Peng Jieru, Shanshan Zhang, Lin Cai, Wencheng Long, Yueshan Wang, Lu Zhang, Yao Dong, Wenqi Zhang, Juan Liao, Chunxia Yang

**Affiliations:** 1 Department of Epidemiology and Biostatistics, West China School of Public Health and West China Fourth Hospital, Sichuan University, Chengdu, 610041 Sichuan China; 2 West China School of Public Health and West China Fourth Hospital, Sichuan University, Chengdu, 610041 Sichuan China; 3 Department of Gastroenterology, West China School of Public Health and West China Fourth Hospital, Sichuan University, Chengdu, 610041 Sichuan China; 4 Non-communicable Diseases Research Center, West China-PUMC C.C. Chen Institute of Health, Sichuan University, Chengdu, 610041 Sichuan China

**Keywords:** Choline intake, Cardiovascular disease, Cancer, Mortality, NHANES

## Abstract

**Background:**

Choline, an indispensable nutrient, plays a pivotal role in various physiological processes. The available evidence regarding the nexus between dietary choline intake and health outcomes, encompassing cardiovascular disease (CVD), cancer, and all-cause mortality, is limited and inconclusive. This study aimed to comprehensively explore the relationship between dietary choline intake and the aforementioned health outcomes in adults aged > 20 years in the U.S.

**Methods:**

This study utilized data from the National Health and Nutrition Examination Survey between 2011 and 2018. Dietary choline intake was evaluated using two 24-h dietary recall interviews. CVD and cancer status were determined through a combination of standardized medical status questionnaires and self-reported physician diagnoses. Mortality data were gathered from publicly available longitudinal Medicare and mortality records. The study utilized survey-weighted logistic and Cox regression analyses to explore the associations between choline consumption and health outcomes. Restricted cubic spline (RCS) analysis was used for dose‒response estimation and for testing for nonlinear associations.

**Results:**

In our study of 14,289 participants (mean age 48.08 years, 47.71% male), compared with those in the lowest quintile (Q1), the adjusted odds ratios (ORs) of CVD risk in the fourth (Q4) and fifth (Q5) quintiles of choline intake were 0.70 (95% CI 0.52, 0.95) and 0.65 (95% CI 0.47, 0.90), respectively (*p* for trend = 0.017). Each 100 mg increase in choline intake was associated with a 9% reduced risk of CVD. RCS analysis revealed a linear correlation between choline intake and CVD risk. Moderate choline intake (Q3) was associated with a reduced risk of mortality, with an HR of 0.75 (95% CI 0.60–0.94) compared with Q1. RCS analysis demonstrated a significant nonlinear association between choline intake and all-cause mortality (*P* for nonlinearity = 0.025). The overall cancer prevalence association was nonsignificant, except for colon cancer, where each 100 mg increase in choline intake indicated a 23% reduced risk.

**Conclusion:**

Elevated choline intake demonstrates an inverse association with CVD and colon cancer, while moderate consumption exhibits a correlated reduction in mortality. Additional comprehensive investigations are warranted to elucidate the broader health implications of choline.

**Supplementary Information:**

The online version contains supplementary material available at 10.1186/s41043-024-00528-0.

## Introduction/background

Cardiovascular disease (CVD) and cancer are two major contributors to global morbidity and mortality and are significant public health challenges worldwide due to changing lifestyles, increasing environment pollution, and aging population. In 2017, noncommunicable causes accounted for a substantial 73.4% of all deaths, resulting in 41.1 million fatalities worldwide, with CVD being responsible for the largest number of deaths (17.8 million), followed by neoplasms (9.56 million) [1]. Some of the factors that contribute to the burden of CVD and cancer are shared including tobacco and alcohol use, physical inactivity and obesity [2]. Identifying modifiable risk factors that influence the development and progression of these diseases is crucial for effective prevention and intervention strategies to further reduce disease-related burden. In recent years, emerging evidence has highlighted the potential role of dietary factors in modulating the risk of both CVD and cancer [3]. For instance, an increased risk of both all-cause mortality and CVD mortality associated with increased egg consumption was confirmed in a high-quality meta-analysis [4].

Choline, an indispensable dietary nutrient found in foods such as eggs, meat, fish, dairy products, and some plant-based sources, has garnered attention for its role in numerous physiological processes, including cell structure and function, neurotransmission, and epigenetic regulation [5]. The existing evidence on the relationship between dietary choline and health outcomes, including CVD, cancer, and mortality, is limited and inconsistent. Research has not conclusively established a clear link between choline intake and the risk of CVD [6], and persistent adherence to a high-choline diet may lower CVD-related mortality by addressing inflammation and associated risk factors [7]. Elevated plasma choline levels are also associated with increased susceptibility to major adverse cardiac events [8], and heightened trimethylamine N-oxide (TMAO), a choline metabolite, has been identified as a potential risk factor for stroke [9]. In the field of oncology, a meta-analysis of eleven scholarly articles indicated a possible inverse correlation between increased choline intake and overall cancer risk [10]. Nonetheless, there are contradictory results concerning colorectal cancer, with some studies revealing negative [11] or no significant relationships [12]. Elevated levels of plasma choline have been positively correlated with increased mortality risk among older adults in the United States [13] and among Chinese adults with hypertension [14]. Moreover, differences in all-cause mortality linked to total choline consumption have been detected among various racial and ethnic groups, including black and white Americans as well as Chinese adults [15]. These findings suggest a possible link between choline intake and health outcomes and indicate a need for further research.

To explore the potential association between dietary choline intake and health-related risks, we conducted a population-based cohort study utilizing data from the National Health and Nutrition Examination Survey (NHANES). Objective evidence is needed to further understand the potential health benefits of choline and the possible mechanisms underlying its involvement. Our primary aim was to comprehensively analyze the link between choline intake and the prevalence of CVD and cancer, as well as disease-specific and all-cause mortality. Additionally, we sought to determine the optimal choline intake for maintaining health and assess the associated health risks linked to inappropriate choline consumption.

## Methods

### Study population

**Fig. 1:**
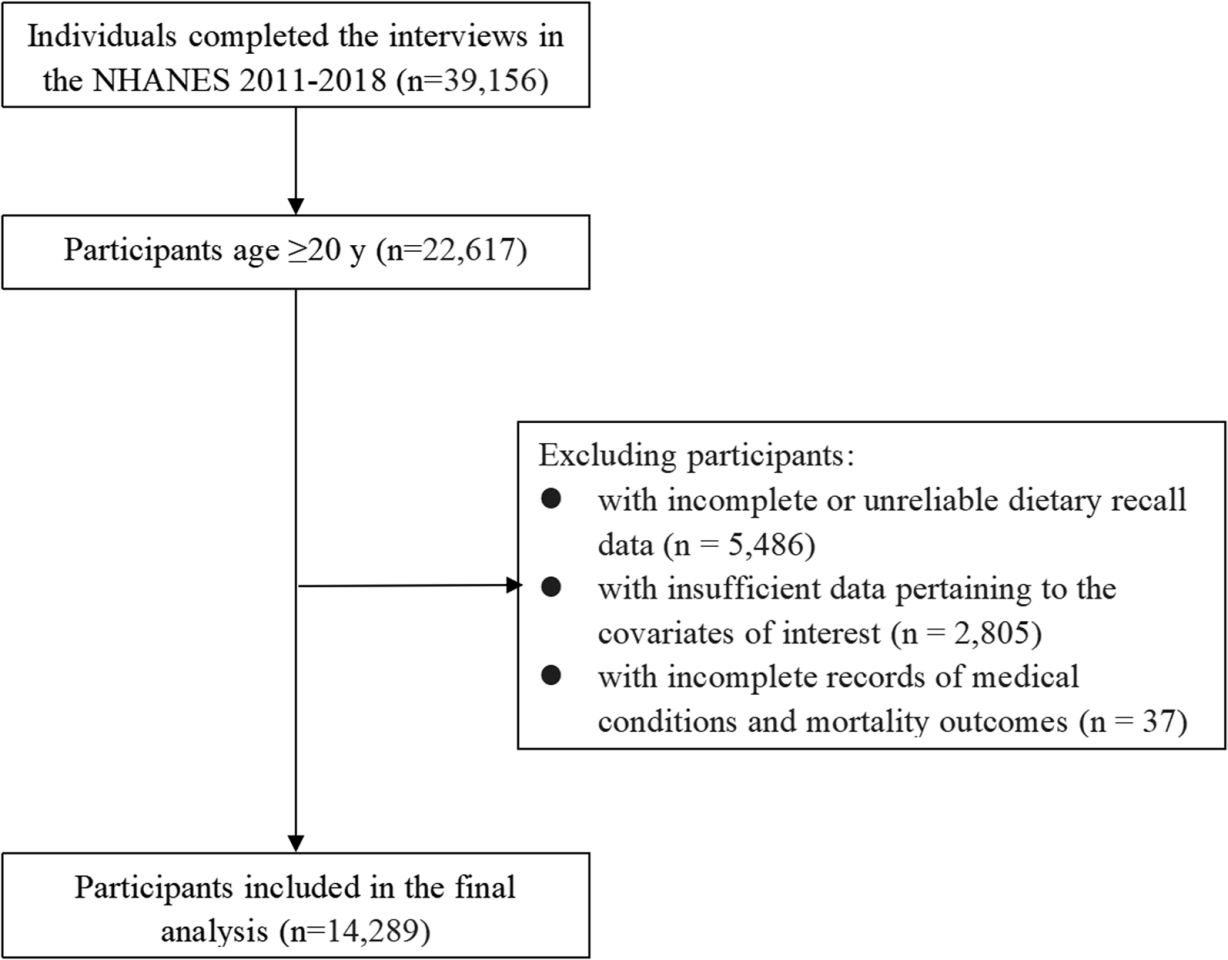
Flow chart of the study. Flow chart of older participants in the National Health and Nutrition Examination Survey 2011–2018 who were included in the analysis between total choline intake and healthy outcomes (n = 14,289)

NHANES is an ongoing project to assess the health and nutritional status of the U.S. civilian population using a rigorous multistage probability sampling design. NHANES protocols were approved by the National Center for Health Statistics Ethics Review Board, which ensures informed consent from all participants. Our investigation used data from four NHANES survey cycles from 2011 to 2018 and included a total of 39,156 participants. We focused on 22,617 individuals aged > 20 years, excluding those with insufficient dietary recall (n = 5486), insufficient covariate data (n = 2805), or incomplete medical and mortality records (n = 37). The final sample size was 14,289 participants. A detailed flowchart of participant selection is shown in Fig. 1.

### Dietary choline intake

Dietary choline intake was assessed for each participant based on the consumption of all foods and beverages during two 24-h dietary recall interviews. In the NHANES, the first dietary interview was conducted in person at the Mobile Examination Center (MEC), followed by a telephone interview 3–10 days later. For our main analysis, we focused on choline intake from the first day of the interview. To increase the robustness of our findings, we calculated average dietary choline intake by pooling data from 2-day recall interviews for further sensitivity analysis.

### Outcome definition

This study focused on the prevalence of self-reported CVD and cancer, as well as all-cause mortality. For CVD assessment, participants were asked if a healthcare professional had diagnosed them with congestive heart failure (CHF), coronary heart disease (CHD), angina pectoris, heart attack, or stroke. An affirmative response classified them as having CVD, and the specific type was determined based on their answers to the five inquiries. Cancer status was determined by participants reporting whether a doctor had previously diagnosed them with cancer. Those who answered "yes" were considered to have cancer, and the specific type of cancer was recorded.

Mortality status was ascertained through publicly available linkage mortality files. The NHANES data from 2011 to 2018 were linked to the Medicare and mortality data. The follow-up duration was calculated from the NHANES examination date to the date of death or December 31, 2019, which marked the end of the follow-up period [16].

### Ascertainment of covariates

Our analysis considered a range of covariates, incorporating demographic factors (age, sex, race/ethnicity: Hispanic, non-Hispanic white, non-Hispanic black, and other), body mass index (BMI), and health-related indicators (educational level, family poverty income ratio (PIR), marital status, smoking status, and physical activity). The dietary variables included total energy intake, dietary folate, vitamin B6, and vitamin B12. Furthermore, clinical conditions, such as high blood pressure, diabetes, and elevated lipid levels, were also included as covariates.

BMI was calculated using measured weight and height at baseline. The family PIR was determined by dividing total family income by the poverty threshold and categorized into three levels (< 1.3, 1.3–3.5, > 3.5). Smoking status was classified as never, former, or current based on cigarette quantity and current status. Physical activity was evaluated through a questionnaire and categorized as low, medium, or optimal following recommended guidelines. The total energy intake and nutrient consumption data were sourced from the complete nutrient file, which contains extensive records of food and beverage consumption during dietary recall. Hypertension was considered present if participants were taking antihypertensive medications or if their blood pressure measurements were equal to or greater than 140/90 mm Hg [17]. Diabetes status was determined through self-reported use of antidiabetic medications and/or by measuring fasting blood glucose and glycosylated hemoglobin levels. A diagnosis of diabetes was established if FBG was 126 mg/dL or higher or if HbA1c was 6.5% or higher [18]. Dyslipidemia was identified in individuals who were taking lipid-lowering medications or had non-high-density lipoprotein cholesterol levels of 160 mg/dL or higher [19].

### Statistical analysis

R software version 4.2.0 was used to conduct the statistical analysis. Sample weights were applied for analyses, addressing the complex survey design and NHANES nonresponse. Continuous variables with a normal distribution are reported as the mean with standard error (SE), nonnormal variables as the median with interquartile range (IQR), and categorical variables as counts and percentages. Dietary choline intake was stratified according to quintile, with quintile 1 (Q1) serving as the reference group (< 20th percentile) and subsequent quintiles labeled Q2 (≥ 20th to 40th percentile), Q3 (≥ 40th to 60th percentile), Q4 (≥ 60th to 80th percentile), and Q5 (≥ 80th percentile).

Survey-weighted logistic regression was employed to evaluate the association between dietary choline intake and the risk of CVD and cancer. Odds ratios (ORs) with 95% confidence intervals (CIs) were estimated. In Model 1, adjustments were made for age, sex, and race/ethnicity. Model 2 expanded the covariates to include educational attainment, marital status, smoking habits, daily energy intake, hypertension, diabetes, and hyperlipidemia. Model 3 further included dietary folate, vitamin B6, and vitamin B12 consumption from Model 2.

Using weighted Cox proportional hazard regression analyses, we investigated the association between dietary choline intake and mortality, controlling for covariates as in the aforementioned logistic regression. Hazard ratios (HRs) and corresponding 95% CIs were calculated. Person-years were computed from participants' recruitment date until mortality occurrence or the end of the follow-up period (December 31, 2019), whichever transpired earlier.

To assess potential nonlinear correlations, we applied restricted cubic spline (RCS) analysis with five knots at the 5th, 25th, 50th, 75th, and 95th percentiles. To minimize outlier influence, we set the reference point at the 25th percentile and confined the analysis between the first and 95th percentiles. Nonlinearity was evaluated using the likelihood ratio test. Receiver operating characteristic (ROC) analysis identified optimal cutoff values for the outcomes.

The analyses were stratified based on age (< 65, ≥ 65 years), sex (male or female), race/ethnicity (Hispanic, non-Hispanic white, non-Hispanic black, and others), and BMI (< 25, 25–30, or < 30 kg/m^2^). Interaction significance was assessed using *p* values from interaction terms involving choline intake and stratification covariates.

To enhance the robustness of our findings, four sensitivity analyses were conducted. These included substituting choline intake with the 2-day average, replacing choline intake with the cumulative sum of first-day supplemental sources and dietary choline intake, repeating primary analyses based on choline intake quartiles, and excluding participants with mortality within a 2-year follow-up interval.

Considering the multistage and complex sampling design employed by the NHANES, survey estimation commands in R Studio, especially the "svyglm" and “svycoxph” functions, were utilized to appropriately consider sampling weights. Two-sided *P* values were used, and *P* < 0.05 indicated statistical significance.

## Results

### Baseline characteristics

According to our analysis of 14,289 participants, 6806 individuals (weighted, 47.71%) were male. The participants had a mean age of 48.08 years (SE, 0.35) and an average daily dietary choline intake of 340.94 mg/day (SE, 2.57). Table 1 presents the baseline characteristics of the participants, categorized into quintiles based on dietary choline intake levels. Individuals in the highest quintile (Q5 group) of choline intake were significantly younger than individuals in the other quartiles were (*P* = 0.010). The proportion of males increased progressively across the quintiles. Participants with higher choline intake were observed to be less predisposed to physical activity and less likely to have a healthy BMI. Nevertheless, they exhibited higher education levels, better economic situations and were more often married.

**Table 1:** Baseline characteristics of participants by quintiles of dietary choline intake, NHANES 2011–2018

Characteristics	Total	Quintile 1	Quintile 2	Quintile 3	Quintile 4	Quintile 5	*P* value^a^
	(N = 14,289)	(N = 3130)	(N = 2804)	(N = 2817)	(N = 2755)	(N = 2783)	
Age, mean (SE), years	48.08 (0.35)	47.83 (0.42)	48.96 (0.57)	48.56 (0.47)	48.56 (0.48)	46.48 (0.47)	0.010
HEI-2015, mean (SE)	53.91 (0.27)	51.43 (0.41)	54.14 (0.42)	54.81 (0.40)	54.62 (0.41)	54.57 (0.43)	< 0.001
Choline intake, mean (SE), mg/day	340.94 (2.57)	130.38 (1.10)	220.28 (0.58)	300.81 (0.74)	403.54 (0.83)	649.54 (5.96)	< 0.001
Total energy intake, mean (SE), calories/day	2094.32 (10.23)	1476.21 (14.00)	1847.56 (13.46)	2081.97 (14.88)	2296.47 (17.51)	2768.96 (26.25)	< 0.001
VB6 intake, mean (SE), mg/day	2.16 (0.02)	1.42 (0.03)	1.82 (0.03)	2.14 (0.03)	2.41 (0.03)	2.99 (0.06)	< 0.001
VB12 intake, mean (SE), mcg/day	4.97 (0.06)	2.95 (0.06)	4.01 (0.08)	4.93 (0.12)	5.61 (0.09)	7.36 (0.19)	< 0.001
Folate intake, mean (SE), mcg/day	401.87 (3.31)	285.35 (3.98)	357.30 (5.05)	414.47 (5.23)	441.19 (5.68)	510.98 (8.22)	< 0.001
*Sex* (%)							< 0.001
Male	6806 (47.71)	924 (27.68)	1117 (37.86)	1299 (45.11)	1527 (56.88)	1939 (70.99)	
Female	7483 (52.29)	2206 (72.32)	1687 (62.14)	1518 (54.89)	1228 (43.12)	844 (29.01)	
*Race* (%)							< 0.001
Hispanic	3246 (13.52)	644 (12.63)	567 (11.47)	655 (13.47)	628 (13.46)	752 (16.56)	
Non-Hispanic white	5797 (68.39)	1240 (66.00)	1190 (70.73)	1138 (69.3)	1154 (69.13)	1075 (66.81)	
Non-Hispanic black	3150 (10.28)	798 (13.01)	647 (10.49)	601 (9.86)	557 (9.27)	547 (8.75)	
Others	2096 (7.81)	448 (8.36)	400 (7.31)	423 (7.37)	416 (8.15)	409 (7.88)	
*Smoking* (%)							< 0.001
Never	11,260 (79.64)	2440 (77.42)	2240 (81.25)	2282 (82.12)	2213 (81.26)	2085 (76.14)	
Former	356 (2.74)	67 (2.30)	59 (2.34)	74 (3.05)	67 (2.78)	89 (3.23)	
Current	2673 (17.62)	623 (20.28)	505 (16.41)	461 (14.82)	475 (15.95)	609 (20.63)	
*Physical activity* (%)							< 0.001
Low	8833 (66.24)	1744 (60.9)	1658 (63.47)	1745 (65.14)	1789 (68.7)	1897 (73.01)	
Medium	1960 (13.07)	454 (13.59)	400 (13.64)	393 (14.46)	366 (12.16)	347 (11.53)	
Optimal	3496 (20.68)	932 (25.51)	746 (22.88)	679 (20.4)	600 (19.15)	539 (15.46)	
*Body mass index* (%)							< 0.001
< 25 kg/m^2^	3957 (27.83)	861 (28.05)	796 (31.11)	792 (27.98)	747 (26.14)	761 (25.87)	
25–30 kg/m^2^	4529 (32.16)	961 (31.47)	877 (30.34)	865 (31.35)	885 (31.80)	941 (35.84)	
> 30 kg/m^2^	5803 (40.01)	1308 (40.48)	1131 (38.55)	1160 (40.67)	1123 (42.06)	1081 (38.29)	
*Education* (%)							< 0.001
Less than high school	2660 (12.07)	704 (15.53)	530 (12.42)	467 (10.45)	437 (10.2)	522 (11.73)	
High school	3194 (22.33)	722 (24.45)	615 (21.45)	621 (21.39)	610 (22.24)	626 (22.13)	
College or higher	8435 (65.60)	1704 (60.02)	1659 (66.13)	1729 (68.15)	1708 (67.56)	1635 (66.14)	
*Family PIR* (%)							< 0.001
< 1.3	2968 (13.72)	764 (17.74)	605 (14.00)	538 (12.10)	515 (12.04)	546 (12.73)	
1.3–3.5	7566 (48.50)	1707 (51.60)	1478 (48.80)	1493 (47.97)	1431 (47.2)	1457 (46.93)	
> 3.5	3755 (37.78)	659 (30.66)	721 (37.20)	786 (39.93)	809 (40.76)	780 (40.34)	
*Marital status* (%)							< 0.001
Married	7390 (56.4)	1434 (50.29)	1451 (55.94)	1490 (57.92)	1529 (60.47)	1486 (57.37)	
Separated	4241 (26.05)	1077 (30.65)	839 (26.62)	815 (24.62)	755 (24.28)	755 (24.08)	
Never married	2658 (17.55)	619 (19.06)	514 (17.44)	512 (17.46)	471 (15.25)	542 (18.56)	
Diabetes (%)	1746 (8.79)	389 (8.57)	328 (7.73)	322 (8.33)	367 (10.4)	340 (8.93)	0.096
Hypertension (%)	9008 (59.51)	1924 (56.47)	1770 (58.01)	1776 (59.97)	1765 (61.56)	1773 (61.54)	0.018
Hypercholesterolemia (%)	1650 (12.13)	415 (14.53)	311 (11.78)	330 (12.65)	291 (10.65)	303 (11.03)	0.017

HEI, Healthy Eating Index; PIR, poverty-to-income ratio; SE, standard error; VB6, Vitamin B-6; VB12, Vitamin B-12

^a^*P* values were calculated using Rao–Scott Chi-square test and analysis of variance for categorical and continuous variables, respectively

### Associations between dietary choline and CVD risk

**Fig. 2:**
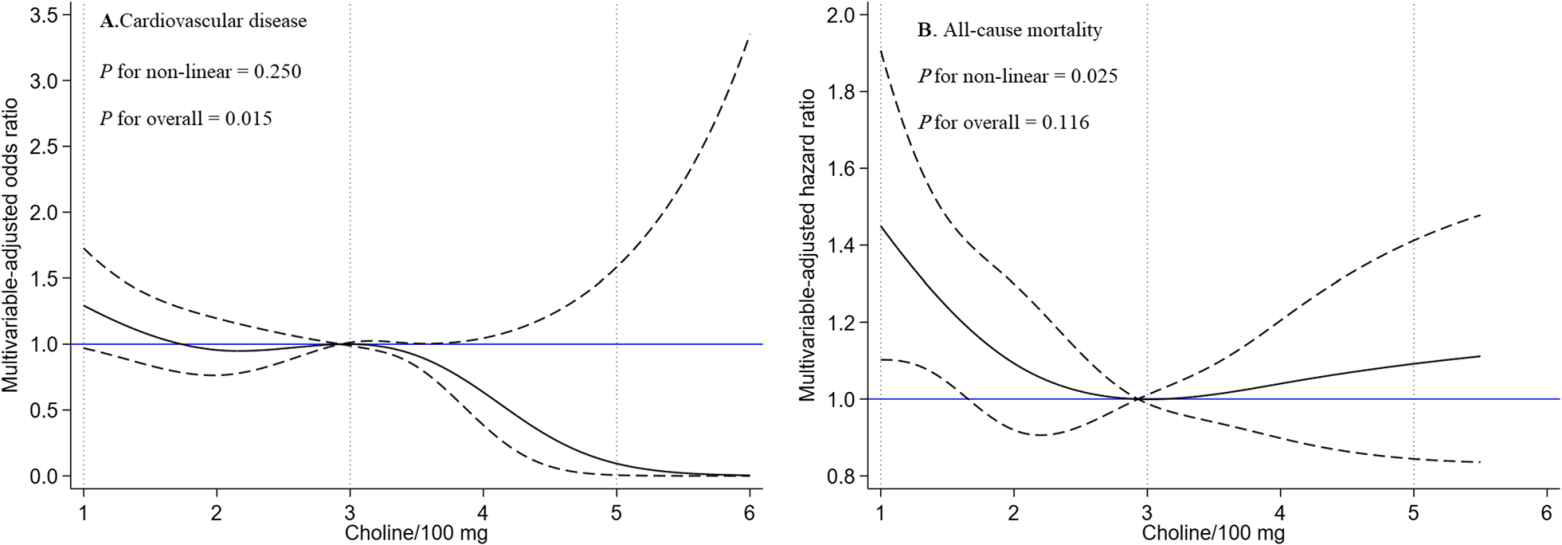
Restricted cubic spline analysis of choline intake and the presence of cardiovascular disease and mortality. *Note*: Fully adjusted restricted cubic spline analysis depicting the association between choline intake and the prevalence of cardiovascular disease and all-cause mortality, utilizing five knots positioned at the fifth, twenty-fifth, fiftieth, seventy-fifth, and ninety-fifth percentiles, with the twenty-fifth percentile serving as the reference point. **A** Association between choline intake (per 100 mg) and CVD prevalence. **B** Association between choline intake (per 100 mg) and all-cause mortality

Table 2 presents the associations between dietary choline consumption and CVD risk, employing both categorical and continuous variables in survey-weighted logistic regression models. CVD prevalence across choline intake quintiles was 11.6%, 10.4%, 9.7%, 9.5%, and 7.9%, respectively. After adjusting for age and sex, individuals in the two highest choline intake quintiles (Q4 and Q5) exhibited significantly lower CVD risk than did those in the lowest quintile (Q1). This association remained significant after further adjustment in Model 2 and Model 3. According to the fully adjusted models, the adjusted odds ratios (95% CIs) for Q4 and Q5 were 0.70 (0.52–0.95) and 0.65 (0.47–0.90), respectively, which was supported by a significant trend (*p* for trend = 0.017). Treating choline as a continuous variable, each 100 mg increase resulted in a 9% decrease in CVD risk (OR = 0.91; 95% CI 0.85–0.99). RCS analysis revealed a significant linear correlation between choline intake and CVD likelihood (*P* for nonlinearity = 0.250, *P* for overall = 0.015). Individuals whose choline intake surpassed the median showed a distinctly lower CVD risk (Fig. 2A). ROC analysis identified 322.5 mg as the optimal cutoff for predicting CVD prevalence. Logistic regression showed that participants with a choline concentration < 322.5 mg had a 1.36 (1.11, 1.68)-fold increased odds of having CVD after covariate adjustment (Additional file 2: Table S1).

**Table 2:** Weighted odds ratios (95% confidence intervals) of CVD and cancer across quintiles of dietary choline intakes, NHANES 2011–2018

Disease	OR (95% CI) by quintile of dietary choline intake, mg/d					Per 100 mg/day increased in choline	*P* for trend
	Quintile 1	Quintile 2	Quintile 3	Quintile 4	Quintile 5		
	(< 182.87)	(182.87 to < 256.89)	(256.89 to < 344.80)	(344.80 to < 472.28)	(≥ 472.28)		
*Cardiovascular disease*							
Cases/participants	364/3130	292/2804	273/2817	261/2755	219/2783	1409/14289	0.017
Model 1	1.00 [Reference]	0.82 (0.67,0.99)	0.82 (0.65,1.04)	0.65 (0.50,0.85)	0.61 (0.47,0.81)	0.91 (0.86,0.98)	
Model 2	1.00 [Reference]	0.88 (0.70,1.11)	0.95 (0.73,1.23)	0.71 (0.52,0.97)	0.67 (0.48,0.93)	0.92 (0.86,0.99)	
Model 3	1.00 [Reference]	0.88 (0.69,1.12)	0.94 (0.72,1.22)	0.70 (0.52,0.95)	0.65 (0.47,0.90)	0.91 (0.85,0.99)	
*Coronary heart disease*							
Cases/participants	138/3130	130/2804	119/2817	101/2755	94/2783	582/14289	0.016
Model 1	1.00 [Reference]	0.92 (0.69,1.24)	0.84 (0.60,1.18)	0.54 (0.39,0.74)	0.64 (0.44,0.93)	0.91 (0.83,0.98)	
Model 2	1.00 [Reference]	0.94 (0.68,1.31)	0.90 (0.61,1.32)	0.50 (0.34,0.75)	0.63 (0.39,1.01)	0.89 (0.81,0.98)	
Model 3	1.00 [Reference]	0.94 (0.67,1.31)	0.90 (0.61,1.33)	0.50 (0.34,0.75)	0.62 (0.38,1.02)	0.89 (0.80,0.98)	
*Cancer*							
Cases/participants	311/3130	276/2804	292/2817	274/2755	221/2783	1374/14289	0.823
Model 1	1.00 [Reference]	0.97 (0.77,1.23)	1.04 (0.83,1.29)	1.17 (0.87,1.56)	0.96 (0.72,1.29)	0.91 (0.94,1.04)	
Model 2	1.00 [Reference]	0.92 (0.70,1.22)	0.98 (0.77,1.24)	1.11 (0.78,1.57)	0.90 (0.62,1.30)	0.99 (0.93,1.05)	
Model 3	1.00 [Reference]	0.93 (0.70,1.22)	0.98 (0.77,1.25)	1.11 (0.78,1.57)	0.90 (0.62,1.30)	0.99 (0.92,1.05)	
*Colon cancer*							
Cases/participants	28/3130	17/2804	15/2817	15/2755	8/2783	83/14289	0.051
Model 1	1.00 [Reference]	1.08 (0.55,2.12)	0.52 (0.23,1.16)	0.88 (0.38,2.03)	0.23 (0.05,0.98)	0.91 (0.63,0.96)	
Model 2	1.00 [Reference]	1.08 (0.55,2.11)	0.52 (0.21,1.28)	0.88 (0.36,2.13)	0.22 (0.05,0.97)	0.77 (0.61,0.97)	
Model 3	1.00 [Reference]	1.08 (0.55,2.13)	0.53 (0.21,1.31)	0.89 (0.36,2.16)	0.23 (0.05,0.96)	0.77 (0.62,0.97)	

CI, confidence interval; NHANES, National Health and Nutrition Examination Survey; OR, odds ratio

Model 1: Logistic regression adjusted for NHANES cycles, age (continuous, year), sex (male and female), race (Hispanic, non-Hispanic white, non-Hispanic black, and others)

Model 2: Adjusted for education (below high school, high school, and college and above), marital status (married, separated, and never married), poverty-to-income ratio (< 1.3, 1.3–3.5, and > 3.5), body mass index (< 25 kg/m^2^, 25–30 kg/m^2^, and > 30 kg/m^2^), daily energy intake (continuous, calories), smoking status (never, ever, and current), alcohol consumption (none, moderate, and heavy), physical activity (light, moderate, and optimal), hypertension (yes and no), diabetes (yes and no), dyslipidemia (yes and no) and covariates adjusted in Model 1

Model 3: Adjusted for dietary folate (mg/day), vitamin B6 (mcg/day), and vitamin B12 (mg/day) consumption, and covariates adjusted in Model 2

To enhance our comprehension, we examined the relationship between choline consumption and specific CVDs, including IHD, CHD, heart disease attacks, and strokes. A notable inverse correlation emerged between total choline intake and CHD (Table 2). Compared with those of the lowest quintile, the ORs for the Q4 and Q5 groups were 0.50 (0.34–0.75) and 0.62 (0.38–1.02), respectively, with a significant trend (*p* for trend = 0.016), emphasizing this association. A 100 mg increase in choline intake was associated with a decreased risk of CHD, as indicated by an OR of 0.89 (0.80–0.98).

No significant associations were observed between choline intake and other categories of CVD in our analysis (Additional file 2: Table S2).

### Associations between dietary choline and cancer risk

As indicated in Table 2, there were no statistically significant associations between choline intake and overall cancer prevalence, whether examined categorically or as a continuous variable. However, a notable finding emerged: for every 100 mg increase in choline intake, the risk of colon cancer was reduced by 23% (OR = 0.77, 95% CI 0.62–0.97) (Table 2). Categorically considering choline intake revealed no significant association with colon cancer prevalence.

### Associations between dietary choline and mortality risk

With a cumulative follow-up time of 71,310 person-years and 809 recorded mortalities, the all-cause mortality rate was 1.13%. After adjusting for potential confounders using survey-weighted Cox regression models, individuals with a moderate level of total choline intake showed a significant reduction in mortality risk (Table 3). The HR (95% CI) for the Q3 group was 0.75 (0.60, 0.94) compared to that for the Q1 group. The RCS analysis revealed a significant nonlinear correlation between choline intake and all-cause mortality (*P* for nonlinearity = 0.025, *P* for overall = 0.117). Those with moderate levels of choline intake exhibited the lowest mortality risk (Fig. 2B).

**Table 3:** Weighted hazard ratios of CVD and all-cause mortality across quintiles of dietary choline intakes, NHANES 2011–2018

Mortality	HR (95% CI) by quintile of dietary choline intake, mg/d					Per 100 mg/day increased in choline	*P* for trend
	Quintile 1	Quintile 2	Quintile 3	Quintile 4	Quintile 5		
	(< 182.87)	(182.87 to < 256.89)	(256.89 to < 344.80)	(344.80 to < 472.28)	(≥ 472.28)		
*All-cause*							
Deaths, No./year	194/15413	170/14006	145/14298	148/13762	152/13831	809/71310	0.725
Model 1	1.00 [Reference]	0.76 (0.57,1.01)	0.65 (0.51,0.81)	0.69 (0.52,0.92)	0.75 (0.58,0.97)	0.97 (0.91,1.03)	
Model 2	1.00 [Reference]	0.78 (0.59,1.02)	0.72 (0.57,0.91)	0.78 (0.58,1.06)	0.85 (0.62,1.17)	0.99 (0.93,1.06)	
Model 3	1.00 [Reference]	0.78 (0.58,1.04)	0.75 (0.60,0.94)	0.80 (0.59,1.09)	0.88 (0.64,1.23)	1.00 (0.92,1.08)	
*CVD*							
Deaths, No./year	62/15413	43/14006	45/14298	49/13762	44/13831	243/71310	0.623
Model 1	1.00 [Reference]	0.53 (0.34,0.83)	0.51 (0.32,0.82)	0.53 (0.32,0.85)	0.53 (0.34,0.83)	1.00 (0.88,1.15)	
Model 2	1.00 [Reference]	0.61 (0.39,0.94)	0.61 (0.37,1.00)	0.65 (0.40,1.05)	0.61 (0.39,0.94)	1.00 (0.86,1.18)	
Model 3	1.00 [Reference]	0.76 (0.47,1.23)	0.78 (0.46,1.33)	0.82 (0.48,1.38)	0.76 (0.47,1.23)	1.03 (0.87,1.20)	
*Cancer*							
Deaths, No./year	35/15413	52/14006	37/14298	34/13762	39/13831	197/71310	0.533
Model 1	1.00 [Reference]	0.74 (0.54,1.01)	0.77 (0.54,1.10)	0.74 (0.5,1.09)	0.58 (0.37,0.9)	0.96 (0.87,1.06)	
Model 2	1.00 [Reference]	0.81 (0.58,1.13)	0.91 (0.61,1.36)	0.88 (0.59,1.33)	0.66 (0.38,1.13)	0.99 (0.88,1.10)	
Model 3	1.00 [Reference]	0.81 (0.58,1.14)	0.92 (0.62,1.38)	0.89 (0.59,1.34)	0.65 (0.38,1.14)	1.00 (0.88,1.15)	

CI, confidence interval; CVD, cardiovascular disease; HR, hazard ratio; NHANES, National Health and Nutrition Examination Survey

Model 1: COX regression adjusted for NHANES cycles, age (continuous, year), sex (male and female), race (Hispanic, non-Hispanic white, non-Hispanic black, and others)

Model 2: Adjusted for education (below high school, high school, and college and above), marital status (married, separated, and never married), poverty-to-income ratio (< 1.3, 1.3–3.5, and > 3.5), body mass index (< 25 kg/m^2^, 25–30 kg/m^2^, and > 30 kg/m^2^), daily energy intake (continuous, calories), smoking status (never, ever, and current), alcohol consumption (none, moderate, and heavy), physical activity (light, moderate, and optimal), hypertension (yes and no), diabetes (yes and no), dyslipidemia (yes and no) and covariates adjusted in Model 1

Model 3: Adjusted for dietary folate (mg/day), vitamin B6 (mcg/day), and vitamin B12 (mg/day) consumption, and covariates adjusted in Model 2

### Stratified and sensitivity analyses

No significant interactions were found between choline intake and demographic factors (age, sex, race/ethnicity, BMI) for CVD risk (*P* values: 0.888, 0.621, 0.743, 0.907), as shown in Additional file 1: Figure S1 A and B.

Consistent findings regarding the association between choline intake and CVD were observed in sensitivity analyses, including 2-day average substitution (Additional file 2: Table S3), incorporating the assessment of first-day supplemental and dietary choline intake (Additional file 2: Table S4), and quartile-based analyses (Additional file 2: Table S5). The preventive effect of moderate choline intake on all-cause mortality was generally significant in Model 1 and Model 2 but lost significance in Model 3 of the sensitivity analysis, which excluded deaths within a 2-year follow-up (Additional file 2: Table S6).

## Discussion

Our nationally representative study of U.S. adults revealed a positive association between higher dietary choline intake and health benefits. There was a substantial correlation between dietary choline intake and CVD, with a particular emphasis on CHD. A choline intake exceeding 322.5 mg per day is notably linked to a significant reduction in CVD risk, emphasizing the potential advantages of increased choline consumption. In addition, moderate choline consumption seems to correlate with decreased mortality risk. Individuals maintaining moderate choline intake, ranging from 256.89 to 344.80 mg/day, exhibit a conspicuously diminished risk of mortality. We did not discern significant links between choline intake and cancer risk, except for colon cancer.

Our study aligns with several previous studies indicating a positive association between increased choline intake and favorable CVD outcomes. Earlier studies underscore the protective role of choline in reducing the risk of stroke [20], lowering CVD incidence [6], mitigating cardiometabolic diseases [21], and reducing the risk of developing hypertension [22]. Evidence also suggests a potential association between higher choline intake and the modulation of inflammatory markers and cardiometabolic parameters [23]. However, a recent systematic review and meta-analysis did not establish clear associations between choline intake and incident CVD [24], and further studies are needed to improve our understanding of these complex associations.

Our study revealed a nonlinear relationship between choline intake and mortality, suggesting that moderate choline consumption is associated with a reduced mortality risk. Interestingly, our findings do not indicate a significant increase in mortality risk with increased choline intake. Similarly, a Japanese cohort study found no substantial evidence supporting a significant association between choline intake and CVD mortality in Japanese men and women [25]. In a prospective study involving diverse racial groups, higher choline intake was associated with increased mortality in black individuals and demonstrated potential associations in the Chinese population. Notably, no significant associations were observed in white participants [15]. In contrast, analysis of data from the NHANES (1999–2010) revealed that individuals in the highest choline consumption quartile faced greater risks of total, cardiovascular disease, and stroke mortality than did those in the lowest quartile [26]. Eggs and milk contribute a relatively higher share to the total choline intake in American dietary habits. Given that eggs are a more concentrated source of choline, they play a significant role in meeting essential choline needs. The frequent consumption of milk further amplifies its significance as a choline resource in the diet [5]. Moderate habitual consumption of eggs and milk should remain a recommendation in the American dietary guidelines, while excess intake of these sources, which may lead to redundant cholesterol and fat, should be discouraged.

Our study did not establish a significant correlation between choline intake and overall cancer risk, which is inconsistent with the findings of a comprehensive meta-analysis of 11 articles that revealed a noteworthy reduction in cancer risk associated with choline consumption [10]. Concerning colon cancer, our results are consistent with prior research, suggesting a potential protective effect of choline intake. Earlier studies, including an Italian multicenter case‒control study [27] and recent observational research [11], have also shown an inverse relationship between choline intake and colorectal cancer risk. Nevertheless, divergent results must be acknowledged; for example, an Iranian case‒control study reported an elevated risk of colorectal cancer with increased choline intake [28], and a large cohort study tracking men over nearly 2 decades found no significant trend in colorectal cancer risk associated with choline intake [12].

The inconsistent findings in studies investigating the impact of dietary choline on health outcomes may stem from variations in the categorization of choline levels. Researchers often classify choline levels into distinct groups based on dietary intake, with the lowest level serving as the reference. This diverse categorization introduces substantial heterogeneity, making direct comparisons challenging. Additionally, the complex nature of choline metabolism, which varies among populations and individuals, may contribute to differences in the effects of dietary choline on health outcomes across studies.

The association between choline levels and health outcomes, including CVD and mortality risk, can be attributed to the involvement of choline in key metabolic processes. Choline is recognized as an essential nutrient and is involved in four primary pathways leading to the synthesis of acetylcholine, betaine, phospholipids, and trimethylamine [29]. These pathways serve essential biological functions. The role of choline includes transformation into acetylcholine for neurotransmission; oxidation to create betaine, which acts as an osmolyte and methyl donor for DNA regulation; and contribution to phosphatidylcholine synthesis. Phosphatidylcholine, a crucial component of the cellular membrane necessary for growth, also participates in cell signaling by generating sphingomyelin, which is vital for myelination in the nervous system [29]. Ensuring adequate choline intake across the lifespan is crucial for maintaining optimal health.

Inadequate choline stores lead to reduced homocysteine methylation, resulting in elevated homocysteine levels. High homocysteine levels are linked to chronic diseases, notably cardiovascular disease and cancer. Adequate choline intake may serve as a mitigating factor against the detrimental effects of elevated homocysteine concentrations, potentially reducing the risk of CVD [30]. The choline and betaine diet-rich diets had the lowest levels of several inflammatory markers, including C-reactive protein (CRP), homocysteine, interleukin-6, and tumor necrosis factor [31].

Upon the consumption of choline-rich foods, specific gut microorganisms can metabolize choline, converting it into trimethylamine, which subsequently enters the bloodstream. Within the liver, trimethylamine undergoes further enzymatic conversion facilitated by flavin-containing monooxygenase 3 (FMO3), a hepatic enzyme. Extensive research has consistently linked elevated TMAO levels to an increased risk of cardiovascular events [32]. However, when dietary choline challenges were introduced, most recent investigations did not report significant increases in chronic TMAO concentrations in the plasma [33]. Given the likely individual variability in TMAO production, which depends on various factors, including one's gut microbiome profile [34], particularly the abundance of the phyla Firmicutes and Proteobacteria [35], as well as liver FMO3 [36], the rate of plasma clearance linked to kidney function [37], and the direct dietary source of TMAO [38], it is essential to consider these multiple determinants.

In a relevant experimental study, compelling evidence regarding the potential cardioprotective mechanisms associated with choline emerged. Choline reduced ventricular dysfunction by altering the expression of proteins involved in ketone body and fatty acid metabolism, as well as inducing a mitochondrial-unfolded protein response; this effect was most likely mediated by activation of the sirtuin 3/AMP-activated protein kinase pathway [39].

### Strengths and limitations

Our study has several strengths. Firstly, it stands as the most extensive analysis to date, providing a comprehensive exploration of the intricate relationship between dietary choline levels and CVD, cancer, and mortality among U.S. adults aged > 20 years. Additionally, the utilization of NHANES data spanning from 2011 to 2018 allows for the precise calculation of dietary nutrient intake by straightforwardly integrating intake data from various sources. This task was challenging due to data collection methods and formatting inconsistencies in earlier releases. Furthermore, our analysis takes into account a wide array of potential confounding factors, thereby enhancing the robustness of our findings. Finally, the use of a nationally representative sample of U.S. adults contributes to the greater generalizability of the results.

Despite these strengths, our study has certain limitations. First, it is essential to acknowledge that data regarding dietary choline levels and covariates collected at baseline may change over time, potentially diminishing the strength of the association between choline levels and health outcomes. Moreover, our categorization of choline levels into quartiles specific to our study population may limit direct comparability with other studies using different cutoff points. Additionally, despite our comprehensive adjustment for potential confounding factors, the presence of residual or unknown confounding variables cannot be entirely ruled out. Finally, given the observational nature of our study design, causal inferences cannot be drawn from our findings.

To improve our understanding of the relationship between dietary choline and health outcomes, further research is needed. Conducting longitudinal studies that track dietary choline levels over time would provide valuable insights into consumption patterns and trends. Standardizing choline level categorizations across studies to improve comparability and enable a more comprehensive assessment of associations on a broader scale. To enhance the validity and reliability of future research findings, it is recommended to conduct thorough analyses to identify and account for potential confounding variables that were not addressed in the current study. Initiating randomized controlled trials could establish causal relationships between dietary choline levels and health outcomes, providing a more robust foundation for evidence-based dietary recommendations.

## Conclusion

In the nationally representative survey encompassing more than 14,000 participants in the U.S., cross-sectional analysis revealed a significant association between high dietary choline levels and a reduced risk of CVD, particularly CHD, as well as colon cancer. Conversely, the cohort study design analysis revealed a nonlinear relationship between choline intake and the risk of mortality, with moderate choline consumption linked to a decreased risk of death. These findings imply that maintaining adequate choline intake may confer substantial health benefits. Moderate consumption of eggs and milk is recommended for Americans to meet essential choline needs. However, it is essential to acknowledge the study's limitations, highlighting the need for further research to comprehensively explore the role of choline in influencing health outcomes in the future.

### Supplementary Information

**Additional file 1:** Supplementary Tables 1-6.

**Additional file 2:** Supplementary Figure 1 (a) and (b)

## Data Availability

The data for this study are already publicly available through the National Center for Health Statistics (NCHS) and National Health and Nutrition Examination Survey (NHANES) websites: https://www.cdc.gov/nchs/nhanes/index.htm.

## Abbreviations

95% CIs: 95% Confidence intervals
BMI: Body mass index
CHD: Coronary heart disease
CHF: Congestive heart failure
CVD: Cardiovascular disease
FMO3: Flavin-containing monooxygenase 3
HRs: Hazard ratios
MEC: Mobile Examination Center
NCHS: National Center for Health Statistics
NHANES: National Health and Nutrition Examination Survey
ORs: Odds ratios
PIR: Poverty-to-income ratio
Q1: Quintile 1
Q2: Quintile 2
Q3: Quintile 3
Q4: Quintile 4
Q5: Quintile 5
RCS: Restricted cubic spline
ROC: Receiver operating characteristic
SE: Standard error
TMAO: Trimethylamine N-oxide
USDA: United States Department of Agriculture
WWEIA: What we eat in America
