# Supplementary material for: Dietary choline intake and health outcomes in U.S. adults: exploring the impact on cardiovascular disease, cancer prevalence, and all-cause mortality
Source: J Health Popul Nutr. 2024 May 6;43:59. doi: 10.1186/s41043-024-00528-0 (PMC11071206; doi:10.1186/s41043-024-00528-0)
Supplement: Supplementary file 1 — Additional file 1: Supplementary Tables 1-6. [file 41043_2024_528_MOESM1_ESM.docx]

**Supplementary Table 1.** Weighted Odds ratios (95% confidence intervals) of CVD and cancer according to the optimal cut-off of dietary choline intakes, NHANES 2011–2018.

| **Dietary choline** | **Cases/participants** | **OR (95% CI)** | | |
| --- | --- | --- | --- | --- |
|  |  | **Model 1** | **Model 2** | **Model 3** |
| **Cardiovascular disease**^a^ |  |  |  |  |
| Equal or above 322.5 mg | 873/8101 | 1.00 [Reference] | 1.00 [Reference] | 1.00 [Reference] |
| Below 322.5 mg | 536/6188 | 1.42 (1.16,1.73) | 1.34 (1.09,1.67) | 1.36 (1.11,1.68) |

Abbreviation: CI, confidence interval; NHANES, National Health and Nutrition Examination Survey; OR, odds ratio.

Model 1: Logistic regression adjusted for NHANES cycles, age (continuous, year), sex (male and female), race (Hispanic, non-Hispanic white, non-Hispanic black, and others);

Model 2: Adjusted for education (below high school, high school, and college and above), marital status (married, separated, and never married), poverty-to-income ratio (<1.3, 1.3-3.5, and >3.5), body mass index (<25 kg/m^2^, 25-30 kg/m^2^, and >30 kg/m^2^), daily energy intake (continuous, calories), smoking status (never, ever, and current), alcohol consumption (none, moderate, and heavy), physical activity (light, moderate, and optimal), hypertension (yes and no), diabetes (yes and no), dyslipidemia (yes and no) and covariates adjusted in Model 1;

Model 3: Adjusted for dietary folate (mg/day), vitamin B6 ( mcg/day), and vitamin B12 (mg/day) consumption, and covariates adjusted in Model 2.

**Supplementary table 2.** Weighted Odds ratios (95% confidence intervals) of CVD subtype according to the quintiles of dietary choline intakes, NHANES 2011–2018.

| **Disease** | **Dietary choline intake, mg/d** | | | | |
| --- | --- | --- | --- | --- | --- |
|  | **Quintile 1** | **Quintile 2** | **Quintile 3** | **Quintile 4** | **Quintile 5** |
|  | **(<205.21)** | **(205.21 to <270.64)** | **(270.64 to <343.66)** | **(343.66 to <442.56)** | **(≥442.56）** |
| **Heart Failure** |  |  |  |  |  |
| Cases/participants | 128/3130 | 93/2804 | 94/2817 | 85/2755 | 71/2783 |
| Model 1 | 1.00 [Reference] | 0.69(0.49,0.97) | 0.88(0.61,1.27) | 0.59(0.41,0.83) | 0.70(0.46,1.05) |
| Model 2 | 1.00 [Reference] | 0.78(0.68,1.31) | 1.07(0.61,1.32) | 0.71(0.34,0.75) | 0.86(0.39,1.01) |
| Model 3 | 1.00 [Reference] | 0.79(0.53,1.19) | 1.09(0.70,1.68) | 0.71(0.47,1.08) | 0.86(0.51,1.47) |
| **Angina** |  |  |  |  |  |
| Cases/participants | 86/3130 | 65/2804 | 68/2817 | 69/2755 | 51/2783 |
| Model 1 | 1.00 [Reference] | 0.75(0.46,1.25) | 0.92(0.57,1.5) | 0.78(0.48,1.26) | 0.75(0.48,1.16) |
| Model 2 | 1.00 [Reference] | 0.80(0.46,1.37) | 1.00(0.61,1.65) | 0.76(0.45,1.26) | 0.72(0.41,1.26) |
| Model 3 | 1.00 [Reference] | 0.80(0.46,1.37) | 1.00(0.60,1.67) | 0.76(0.45,1.27) | 0.72(0.40,1.28) |
| **Heart disease attack** |  |  |  |  |  |
| Cases/participants | 136/3130 | 124/2804 | 116/2817 | 112/2755 | 104/2783 |
| Model 1 | 1.00 [Reference] | 0.97(0.70,1.35) | 0.88(0.63,1.24) | 0.68(0.47,0.98) | 0.84(0.55,1.28) |
| Model 2 | 1.00 [Reference] | 1.09(0.77,1.56) | 1.07(0.77,1.50) | 0.79(0.52,1.20) | 1.02(0.65,1.59) |
| Model 3 | 1.00 [Reference] | 1.10(0.76,1.57) | 1.09(0.78,1.52) | 0.81(0.53,1.21) | 1.03(0.64,1.64) |
| **Stroke** |  |  |  |  |  |
| Cases/participants | 158/3130 | 109/2804 | 100/2817 | 97/2755 | 77/2783 |
| Model 1 | 1.00 [Reference] | 0.74(0.54,1.01) | 0.77(0.54,1.10) | 0.74(0.50,1.09) | 0.58(0.37,0.90) |
| Model 2 | 1.00 [Reference] | 0.81(0.58,1.13) | 0.91(0.61,1.36) | 0.88(0.59,1.33) | 0.66(0.38,1.13) |
| Model 3 | 1.00 [Reference] | 0.81(0.58,1.14) | 0.92(0.62,1.38) | 0.89(0.59,1.34) | 0.65(0.38,1.14) |

Abbreviation: CI, confidence interval; NHANES, National Health and Nutrition Examination Survey; OR, odds ratio.

Model 1: Logistic regression adjusted for NHANES cycles, age (continuous, year), sex (male and female), race (Hispanic, non-Hispanic white, non-Hispanic black, and others);

Model 2: Adjusted for education (below high school, high school, and college and above), marital status (married, separated, and never married), poverty-to-income ratio (<1.3, 1.3-3.5, and >3.5), body mass index (<25 kg/m^2^, 25-30 kg/m^2^, and >30 kg/m^2^), daily energy intake (continuous, calories), smoking status (never, ever, and current), alcohol consumption (none, moderate, and heavy), physical activity (light, moderate, and optimal), hypertension (yes and no), diabetes (yes and no), dyslipidemia (yes and no) and covariates adjusted in Model 1;

Model 3: Adjusted for dietary folate (mg/day), vitamin B6 ( mcg/day), and vitamin B12 (mg/day) consumption, and covariates adjusted in Model 2.

**Supplementary Table 3.** Weighted Odds Ratios (95% Confidence Intervals) for Cardiovascular Disease and Hazard Ratios (95% Confidence Intervals) for All-Cause Mortality across Quintiles of Dietary Choline Intake (Average of First and Second-Day Intake) in NHANES 2011–2018.

| **Disease** | **Dietary choline intake, mg/d** | | | | |
| --- | --- | --- | --- | --- | --- |
|  | **Quintile 1** | **Quintile 2** | **Quintile 3** | **Quintile 4** | **Quintile 5** |
|  | **(<205.25)** | **(205.25 to <270.60)** | **(270.60 to <343.75)** | **(343.75 to <442.55)** | **(≥442.60）** |
| **Cardiovascular disease ^a^** |  | | | | |
| Cases/participants | 364/3130 | 292/2804 | 273/2817 | 261/2754 | 219/2784 |
| Model 1 | 1.00 [Reference] | 0.63 (0.50, 0.79) | 0.82 (0.63, 1.07) | 0.61 (0.46, 0.80) | 0.71 (0.54, 0.93) |
| Model 2 | 1.00 [Reference] | 0.72 (0.56, 0.91) | 0.97 (0.72, 1.31) | 0.71 (0.52, 0.99) | 0.90 (0.65, 1.25) |
| Model 3 | 1.00 [Reference] | 0.72 (0.56, 0.91) | 0.96 (0.72, 1.29) | 0.71 (0.52, 0.97) | 0.88 (0.65, 1.21) |
| **Cancer ^a^** |  |  |  |  |  |
| Cases/participants | 311/3130 | 276/2804 | 292/2817 | 274/2754 | 221/2784 |
| Model 1 | 1.00 [Reference] | 0.98 (0.74, 1.30) | 1.19 (0.90, 1.58) | 1.19 (0.92, 1.54) | 1.01 (0.72, 1.42) |
| Model 2 | 1.00 [Reference] | 0.96 (0.71, 1.29) | 1.17 (0.86, 1.60) | 1.10 (0.79, 1.54) | 0.97 (0.63, 1.51) |
| Model 3 | 1.00 [Reference] | 0.96 (0.71, 1.28) | 1.17 (0.86, 1.60) | 1.10 (0.79, 1.53) | 0.96 (0.61, 1.52) |
| **All-cause mortality ^b^** |  |  |  |  |  |
| Deaths, No./year | 193/15323 | 167/14333 | 150/14175 | 164/13533 | 135/13944 |
| Model 1 | 1.00 [Reference] | 0.68(0.52,0.89) | 0.62(0.48,0.80) | 0.73(0.55,0.98) | 0.70(0.53,0.91) |
| Model 2 | 1.00 [Reference] | 0.79(0.62,1.01) | 0.74(0.56,0.97) | 0.90(0.64,1.26) | 0.91(0.63,1.31) |
| Model 3 | 1.00 [Reference] | 0.80(0.63,1.02) | 0.76(0.58,1.00) | 0.92(0.66,1.29) | 0.96(0.67,1.37) |

Abbreviation: NHANES, National Health and Nutrition Examination Survey.

a: Logistic regression analysis to assess the impact of dietary choline: presentation of odds ratios with 95% confidence intervals in the table.

b: Cox regression analysis to evaluate the effect of dietary choline: presentation of hazard ratios with 95% confidence intervals in the table.

Model 1: Logistic regression adjusted for NHANES cycles, age (continuous, year), sex (male and female), race (Hispanic, non-Hispanic white, non-Hispanic black, and others);

Model 2: Adjusted for education (below high school, high school, and college and above), marital status (married, separated, and never married), poverty-to-income ratio (<1.3, 1.3-3.5, and >3.5), body mass index (<25 kg/m^2^, 25-30 kg/m^2^, and >30 kg/m^2^), daily energy intake (continuous, calories), smoking status (never, ever, and current), alcohol consumption (none, moderate, and heavy), physical activity (light, moderate, and optimal), hypertension (yes and no), diabetes (yes and no), dyslipidemia (yes and no) and covariates adjusted in Model 1;

Model 3: Adjusted for dietary folate (mg/day), vitamin B6 ( mcg/day), and vitamin B12 (mg/day) consumption, and covariates adjusted in Model 2.

Supplementary Table 4: Weighted Odds Ratios (95% Confidence Intervals) for Cardiovascular Disease and Cancer and Hazard Ratios (95% Confidence Intervals) for All-Cause Mortality across Quintiles of Choline Intake (Total of First Day Supplemental and Dietary Intake) in NHANES 2011–2018.

| **Disease** | **Dietary choline intake, mg/d** | | | | |
| --- | --- | --- | --- | --- | --- |
|  | **Quintile 1** | **Quintile 2** | **Quintile 3** | **Quintile 4** | **Quintile 5** |
|  | **(<184.30)** | **(184.30 to <259.60)** | **(259.60 to <345.60)** | **(345.70 to <474.00)** | **(≥474.00）** |
| **Cardiovascular disease ^a^** |  |  |  |  |  |
| Cases/participants | 355/3015 | 272/2689 | 271/2646 | 243/2627 | 207/2643 |
| Model 1 | 1.00 [Reference] | 0.76(0.62,0.92) | 0.86(0.68,1.09) | 0.61(0.47,0.8) | 0.60(0.46,0.79) |
| Model 2 | 1.00 [Reference] | 0.82(0.65,1.04) | 1.02(0.78,1.34) | 0.68(0.5,0.91) | 0.69(0.50,0.95) |
| Model 3 | 1.00 [Reference] | 0.82(0.64,1.04) | 1.01(0.77,1.33) | 0.67(0.5,0.89) | 0.67(0.49,0.92) |
| **Cancer ^a^** |  |  |  |  |  |
| Cases/participants | 300/3015 | 267/2689 | 275/2646 | 266/2627 | 206/2643 |
| Model 1 | 1.00 [Reference] | 0.97(0.75,1.24) | 1.03(0.82,1.30) | 1.18(0.87,1.60) | 0.91(0.67,1.24) |
| Model 2 | 1.00 [Reference] | 0.91(0.68,1.22) | 0.96(0.74,1.23) | 1.11(0.77,1.59) | 0.83(0.56,1.24) |
| Model 3 | 1.00 [Reference] | 0.92(0.68,1.23) | 0.96(0.74,1.24) | 1.11(0.77,1.59) | 0.83(0.55,1.24) |
| **All-cause mortality ^b^** |  |  |  |  |  |
| Deaths, No./year | 187/14809 | 167/13404 | 133/13537 | 138/13140 | 147/13137 |
| Model 1 | 1.00 [Reference] | 0.79(0.59,1.07) | 0.59(0.46,0.76) | 0.70(0.53,0.92) | 0.78(0.60,1.01) |
| Model 2 | 1.00 [Reference] | 0.79(0.59,1.07) | 0.67(0.51,0.86) | 0.78(0.58,1.03) | 0.87(0.63,1.20) |
| Model 3 | 1.00 [Reference] | 0.80(0.59,1.08) | 0.68(0.53,0.88) | 0.80(0.59,1.06) | 0.91(0.65,1.26) |

Abbreviation: NHANES, National Health and Nutrition Examination Survey.

a: Logistic regression analysis to assess the impact of dietary choline: presentation of odds ratios with 95% confidence intervals in the table.

b: Cox regression analysis to evaluate the effect of dietary choline: presentation of hazard ratios with 95% confidence intervals in the table.

Model 1: Logistic regression adjusted for NHANES cycles, age (continuous, year), sex (male and female), race (Hispanic, non-Hispanic white, non-Hispanic black, and others);

Model 2: Adjusted for education (below high school, high school, and college and above), marital status (married, separated, and never married), poverty-to-income ratio (<1.3, 1.3-3.5, and >3.5), body mass index (<25 kg/m^2^, 25-30 kg/m^2^, and >30 kg/m^2^), daily energy intake (continuous, calories), smoking status (never, ever, and current), alcohol consumption (none, moderate, and heavy), physical activity (light, moderate, and optimal), hypertension (yes and no), diabetes (yes and no), dyslipidemia (yes and no) and covariates adjusted in Model 1;

Model 3: Adjusted for dietary folate (mg/day), vitamin B6 ( mcg/day), and vitamin B12 (mg/day) consumption, and covariates adjusted in Model 2.

Supplementary Table 5: Weighted Odds Ratios (95% Confidence Intervals) for Cardiovascular Disease and Hazard Ratios (95% Confidence Intervals) for All-Cause Mortality across Quartiles of Dietary Choline Intake in NHANES 2011–2018.

| **Disease** | **Dietary choline intake, mg/d** | | | |
| --- | --- | --- | --- | --- |
|  | **Quartile 1** | **Quartile 2** | **Quartile 3** | **Quartile 4** |
|  | **(<201.60)** | **(201.60 to <300.20)** | **(300.20 to <432.70)** | **(≥432.70)** |
| **Cardiovascular disease ^a^** |  |  |  |  |
| Cases/participants | 429/3847 | 368/3528 | 330/3458 | 282/3455 |
| Model 1 | 1.00 [Reference] | 0.81(0.66,1.00) | 0.77(0.60,0.98) | 0.66(0.51,0.84) |
| Model 2 | 1.00 [Reference] | 0.89(0.7,1.12) | 0.85(0.65,1.12) | 0.73(0.55,0.97) |
| Model 3 | 1.00 [Reference] | 0.89(0.7,1.12) | 0.85(0.65,1.10) | 0.72(0.54,0.94) |
| **Cancer ^a^** |  |  |  |  |
| Cases/participants | 370/3847 | 356/3528 | 363/3458 | 285/3455 |
| Model 1 | 1.00 [Reference] | 0.99(0.81,1.21) | 1.17(0.93,1.47) | 0.97(0.74,1.28) |
| Model 2 | 1.00 [Reference] | 0.94(0.75,1.17) | 1.12(0.86,1.45) | 0.91(0.65,1.28) |
| Model 3 | 1.00 [Reference] | 0.94(0.75,1.18) | 1.12(0.86,1.45) | 0.90(0.65,1.27) |
| **All-cause mortality ^b^** |  |  |  |  |
| Deaths, No./year | 233/18998 | 201/17578 | 186/17588 | 189/17143 |
| Model 1 | 1.00 [Reference] | 0.76(0.58,0.99) | 0.75(0.59,0.94) | 0.76(0.61,0.96) |
| Model 2 | 1.00 [Reference] | 0.81(0.62,1.06) | 0.87(0.69,1.09) | 0.89(0.68,1.16) |
| Model 3 | 1.00 [Reference] | 0.82(0.62,1.09) | 0.89(0.71,1.12) | 0.92(0.70,1.20) |

Abbreviation: NHANES, National Health and Nutrition Examination Survey.

a: Logistic regression analysis to assess the impact of dietary choline: presentation of odds ratios with 95% confidence intervals in the table.

b: Cox regression analysis to evaluate the effect of dietary choline: presentation of hazard ratios with 95% confidence intervals in the table.

Model 1: Logistic regression adjusted for NHANES cycles, age (continuous, year), sex (male and female), race (Hispanic, non-Hispanic white, non-Hispanic black, and others);

Model 2: Adjusted for education (below high school, high school, and college and above), marital status (married, separated, and never married), poverty-to-income ratio (<1.3, 1.3-3.5, and >3.5), body mass index (<25 kg/m^2^, 25-30 kg/m^2^, and >30 kg/m^2^), daily energy intake (continuous, calories), smoking status (never, ever, and current), alcohol consumption (none, moderate, and heavy), physical activity (light, moderate, and optimal), hypertension (yes and no), diabetes (yes and no), dyslipidemia (yes and no) and covariates adjusted in Model 1;

Model 3: Adjusted for dietary folate (mg/day), vitamin B6 ( mcg/day), and vitamin B12 (mg/day) consumption, and covariates adjusted in Model 2.

Supplementary Table 6: Weighted Hazard Ratios (95% Confidence Intervals) for All-Cause Mortality across Quintiles of Dietary Choline Intake in NHANES 2011–2018 except for participants who died within 2 years of follow-up.

| **Disease** | **Dietary choline intake, mg/d** | | | | |
| --- | --- | --- | --- | --- | --- |
|  | **Quintile 1** | **Quintile 2** | **Quintile 3** | **Quintile 4** | **Quintile 5** |
|  | **(<184.30)** | **(184.30 to <260.50)** | **(260.50 to <346.70)** | **(346.80 to <476.50)** | **(≥476.70）** |
| **All-cause mortality** |  |  |  |  |  |
| Deaths, No./year | 140/15019 | 119/13664 | 105/13557 | 111/13234 | 101/13049 |
| Model 1 | 1.00 [Reference] | 0.78(0.54,1.14) | 0.67(0.50,0.90) | 0.81(0.61,1.07) | 0.79(0.58,1.07) |
| Model 2 | 1.00 [Reference] | 0.80(0.55,1.16) | 0.75(0.55,1.03) | 0.89(0.65,1.21) | 0.89(0.60,1.30) |
| Model 3 | 1.00 [Reference] | 0.80(0.55,1.17) | 0.77(0.56,1.05) | 0.91(0.66,1.24) | 0.91(0.62,1.34) |

Abbreviation: CI, confidence interval; CVD, cardiovascular disease; HR, hazard ratio; NHANES, National Health and Nutrition Examination Survey.

Model 1: COX regression adjusted for NHANES cycles, age (continuous, year), sex (male and female), race (Hispanic, non-Hispanic white, non-Hispanic black, and others);

Model 2: Adjusted for education (below high school, high school, and college and above), marital status (married, separated, and never married), poverty-to-income ratio (<1.3, 1.3-3.5, and >3.5), body mass index (<25 kg/m^2^, 25-30 kg/m^2^, and >30 kg/m^2^), daily energy intake (continuous, calories), smoking status (never, ever, and current), alcohol consumption (none, moderate, and heavy), physical activity (light, moderate, and optimal), hypertension (yes and no), diabetes (yes and no), dyslipidemia (yes and no) and covariates adjusted in Model 1;

Model 3: Adjusted for dietary folate (mg/day), vitamin B6 ( mcg/day), and vitamin B12 (mg/day) consumption, and covariates adjusted in Model 2.

Supplementary Figure 1: Stratified Analysis of Age, Sex, Race, and Body Mass Index (BMI) with Adjustments for Various Covariates.

A. The stratified analysis is stratified by age (<60 years old and ≥60 years old) and body mass index (<25, 25-30, >30 kg/m^2^).

B. The stratified analysis is stratified by sex (male and female) and race (Hispanic, non-Hispanic white, non-Hispanic black, and others).

Adjustments were made for education (below high school, high school, and college and above), marital status (married, separated, and never married), poverty-to-income ratio (<1.3, 1.3-3.5, and >3.5), daily energy intake (continuous, calories), smoking status (never, ever, and current), alcohol consumption (none, moderate, and heavy), physical activity (light, moderate, and optimal), hypertension (yes and no), diabetes (yes and no), dyslipidemia (yes and no), dietary folate (mg/day), vitamin B6 (mcg/day), and vitamin B12 (mg/day).
