# Supplementary figures and images for: Dietary choline intake and health outcomes in U.S. adults: exploring the impact on cardiovascular disease, cancer prevalence, and all-cause mortality
Source: J Health Popul Nutr. 2024 May 6;43:59. doi: 10.1186/s41043-024-00528-0 (PMC11071206; doi:10.1186/s41043-024-00528-0)

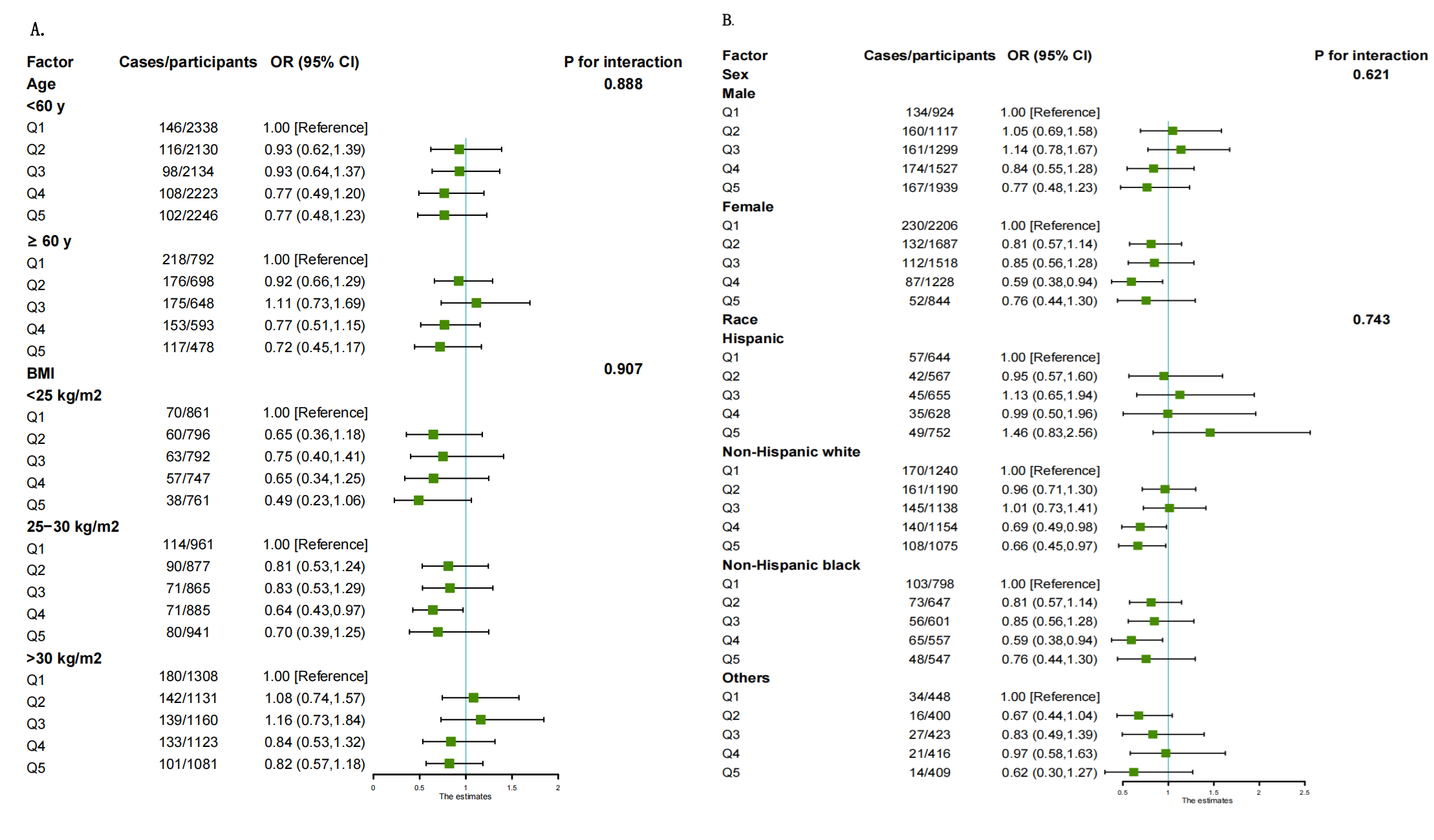

Supplement: Supplementary file 2 — Additional file 2: Supplementary Figure 1 (a) and (b) [file 41043_2024_528_MOESM2_ESM.png]
